# Generation and Characterization of an Osteoclastic Cell Model of Gaucher’s Bone Disease

**DOI:** 10.1007/s00223-026-01616-5

**Published:** 2026-10-01

**Authors:** Juan-David Patiño-Salazar, Juan Diego Gutiérrez-Ávila, Sara Heredero-Jimenez, Eduardo Martín-Guerrero, Núria Martínez-Gil, Mónica Cozar, Diana Ovejero, Xavier Nogués, Laura Cinque, Juan A. Ardura, Arancha R. Gortazar, Daniel Grinberg, Susanna Balcells, Natalia Garcia-Giralt, Raquel Rabionet

**Affiliations:** 1 Department of Genetics, Microbiology and Statistics, Faculty of Biology, University of Barcelona, CIBERER, IBUB, IRSJD, Barcelona, Spain; 2 Bone Physiopathology Laboratory, Institute of Applied Molecular Medicine (IMMA), San Pablo-CEU University, CEU Universities, Campus Montepríncipe, Madrid, Spain; 3 Musculoskeletal Research Group, Hospital del Mar Research Institute, CIBERFES, ISCIII, Barcelona, Spain; 4 Department of Clinical Medicine and Surgery, Telethon Institute of Genetics and Medicine (TIGEM), Federico II University, Naples, Italy; 5 Present Address: Department of Clinical and Molecular Genetics, Vall d’Hebron University Hospital, Medical Genetics Group, Vall d’Hebron Research Institute, Barcelona, Spain

**Keywords:** Gaucher disease, *Gba1* Knockout, Osteoclasts

## Abstract

Gaucher disease (GD) is caused by recessive mutations in the *GBA1* gene (Glucosylceramidase beta 1) generating lysosomal accumulation of glucosylceramides. Bone phenotypes related to GD include Erlenmeyer flask deformity, osteosclerosis, osteonecrosis, osteopenia, and bone pain. Lysosomes modulate osteoclast differentiation, playing a fundamental role in osteoclastogenesis and bone resorption. However, the role of *GBA1* mutations in the osteoclast function remains poorly understood. We aimed to generate and characterize an in vitro osteoclast cell model of GD. The *Gba1* gene was knocked out with CRISPR/Cas9 in the murine macrophage cell line RAW264.7. One RAW-Gba1KO clone displaying no GBA1 enzymatic activity was selected. Non-edited RAW264.7 cells were used as controls (wt). Expression of osteoclast biomarkers, *Rank*, *Trap*, *Nfatc1*, and pro-cathepsin K/cathepsin K as well as activity of TRAP protein and bone resorption were assessed. RAW-Gba1KO cells showed reduced expression of the osteoclast differentiation markers Rank and Nfatc1, suggesting that osteoclast differentiation is delayed or impaired compared with wt cells. Despite this, KO cells exhibited higher expression of Trap and cathepsin K, indicating enhanced osteoclast activity. Functional assays confirmed this finding, showing significantly increased TRAP activity and bone resorption in KO cells during differentiation. Additionally, KO cells displayed elevated levels of cathepsin K and pro-cathepsin K, particularly before differentiation, which may contribute to the increased TRAP activation. In conclusion, lack of Gba1 activity induced changes in our osteoclast model which enhanced its bone resorption potential, demonstrating a major intrinsic osteoclastic defect in Gaucher disease of bone.

**Supplementary Information:**

The online version contains supplementary material available at https://doi.org/10.1007/s00223-026-01616-5.

## Introduction

Gaucher disease (GD, OMIM #230800, ORPHA355) is a rare autosomal recessive disorder caused by biallelic pathogenic variants in the *GBA1* gene (GRCh37/hg19 Chromosome 1: 155,204,239 to 155,214,653), leading to deficient activity of glucocerebrosidase (GCase; EC 3.2.1.45; GBA1) which generates accumulation of glucosylceramide and glucosylsphingosine in lysosomes of the monocyte/macrophage system [1]. Hence, because osteoclasts derive from monocyte/macrophage precursors, they are directly affected.

Although the phenotype of Gaucher disease is variable, three clinical forms have been identified according to the severity of neuronopathic involvement, namely: non-neuronopathic (GD1, OMIM 230800); acute neuronopathic (GD2, OMIM 230900); and chronic or subacute neuronopathic (GD3, OMIM 231000) [2, 3].

GD1 is the most common form, presenting with hepatosplenomegaly, pancytopenia, and extensive bone involvement (e.g., osteonecrosis, fractures, osteolytic lesions, Erlenmeyer flask deformity), often leading to disability and reduced quality of life. GD2 is a severe, early-onset form characterized by rapid neurologic decline, hepatosplenomegaly, and death usually by age 2. GD3 is the subacute form of neuronopathic Gaucher disease. It has later onset and slower progression compared to the acute form of GD2 [4, 5].

There are some interesting genotype-phenotype correlations that should be mentioned. The N370S mutation (p.Asn409Ser), one of the most prevalent GD mutations, causes GD1, while L444P (p.Leu483Phe), another prevalent mutation, is present in patients with GD2 or GD3. Bearing one allele with the N370S mutation is sufficient to present with a non-neuronopathic disease, independently of the mutation in the other allele. The N370S mutation (and a few other variants) present in GD1 patients give rise to a GBA enzyme with a relatively high residual activity [6].

GD1 presents skeletal symptoms in up to 80% of patients, which significantly reduces their quality of life. Bone involvement in GD may also include medullary and corticocancellous infarcts, pathological fractures, osteonecrosis of humeral or femoral heads, and collapse of vertebrae [7–9].

Lysosomes modulate osteoclast differentiation, playing a fundamental role in osteoclastogenesis and bone resorption [10]. In a previous work, a *GBA1* knockout monocytic cell line (GBAKO-THP1) was generated demonstrating an increased osteoclastogenesis [11]. However, the precise molecular mechanisms underlying the osteoclast function in GD are still unclear.

In this study, we aim to produce an osteoclast model with an inactivated *Gba1* gene using CRISPR/Cas9 and to evaluate its intrinsic performance to broaden our understanding of the bone phenotype in GD.

## Materials and Methods

### Generation of an Osteoclast-Like Cell Model with CRISPR/Cas9

The mouse macrophage cell line RAW 264.7 (ATCC, Manassas, VA, USA) was used as a model of osteoclast-like cells. *Gba1* editing was performed at the Organelle Homeostasis Laboratory of TIGEM (Naples, Italy) using the pSpCas9(BB)-2 A-GFP (PX458) plasmid and Sigma-Aldrich Synthetic guide RNA (RN00064-0684536) (sgRNA) (Table S1) which targets exon 4 of the mouse *Gba1* gene. For guide selection, off-target site prediction was carried out using Benchling (https://www.benchling.com/) (Table S2). The sgRNA was cloned into the pSpCas9(BB)-2 A-GFP vector following the protocol of Ran et al. 2013 [12]. After the sgRNA was cloned and tested, the cells were seeded one day before transfection in a 6-well plate (3 × 10^5^ cell/well). Reverse transfection was performed using 2500 ng of pSpCas9 plasmid containing sgRNA, 7.5 µL of Lipofectamine™ 3000 and 5 µL of Lipofectamine™ P3000 reagent (Invitrogen-Thermo Fisher Scientific, USA) according to the manufacturer’s protocols. After 48–72 h the edited pool was sorted by single-cell sorting in flow cytometry (BD Biosciences LSR II flow cytometer, San Jose, CA, USA). From a single clone, cells were cultured to perform DNA extraction to identify edited clones by Sanger sequencing (Supplementary Figure S1). Edited clones predicted to lead to knock out (KO) proteins were analysed by western blot (WB) to confirm lack of glucocerebrosidase (GCase) expression. Non-edited RAW cells were used as a wt for comparisons with KO cells.

### Cell Cultures

RAW wt and RAW-*Gba1*KO cells were cultured and maintained in DMEM medium (Gibco) with 10% fetal bovine serum (FBS) and 1% penicillin/streptomycin (complete medium), in a humidified atmosphere containing 5% CO_2_ at 37 °C (basal state).

### Glucocerebrosidase Enzymatic Activity

Glucocerebrosidase (GCase) activity was measured using the fluorogenic substrate 4-methylumbelliferyl–d-glucopyranoside (Sigma-Aldrich, St. Louis, MO, USA). The total amount of protein in cell lysates was measured using the Bradford assay (BioRad, Hercules, CA, USA), following manufacturer’s instructions. In each well of a 96 well plate, 24 µL containing 24 µg of protein was incubated at 37 °C for 30 min. After that, 100 µL of McIlvaine’s buffer (Citrate-Na_2_HPO_4_ solution 150 mM; Taurocholate 0,2%; Triton X-100 0,1%; Bovine serum albumin (BSA) 0,1%; substrate 1.25 mg/mL; pH 5.2) were added and the plate was incubated again at 37 °C for 30 min. The reaction was stopped with NaOH-glycine buffer 0.3 M pH 10.4 and the fluorescent product was quantified using a fluorimeter (Thermo Scientific Fluoroskan Ascent FL Microplate Fluorimeter and Luminometer) at an excitation wavelength of 365 nm and emission of 495 nm. Quantification was performed in comparison to a reading without adding substrate to the cells (blank). Readings lower than the blank are shown as 0. This assay was performed once for each selected clone.

### Osteoclast Differentiation Assays

To induce osteoclastogenesis, RAW wt and RAW-*Gba1*KO cells were seeded at a density of 25,000 cells/cm^2^ in 6-well plates. After 48 h, the DMEM complete medium was replaced by MEM-alpha medium (Gibco) containing 1% FBS, 1% penicillin/streptomycin, and 50 ng/mL of recombinant murine sRANK ligand (RANKL) (PeproTech) for up to 8 days, replacing the media every 48 h. Osteoclast maturation was checked at four days (Supplementary figure S2) and eight days of differentiation (Supplementary figure S3). Three independent replicates were carried out for each differentiation assay.

### Characterization of Lysosomes

Lysosomes of RAW (wt and KO) cells were determined by flow cytometry using LysoTracker™ Green DND-26 (Thermo Fisher Scientific) in live cells cultured for 7 days. Briefly, 7 × 10^5^ cells were resuspended in 15 mL tubes containing 6 mL of complete medium supplemented with Lysotracker Green 50nM. Cells were incubated at 37°C, 5% CO_2_ for 5 min in the dark and then washed with phosphate-buffered saline 1X (PBS, PanReac AppliChem). Immediately thereafter, the intensity of fluorescence of lysosomes was measured with a Gallios (Beckman Coulter) flow cytometer of the Scientific and Technological Centers of the Universitat de Barcelona. Four to six independent replicates were performed.

### RT-PCR

RAW (wt and KO) cells were seeded at a density of 7 × 10^5^ in 6-well plates. After 48 h for basal state determinations (undifferentiated cells) and 8 days for differentiation analysis (after adding 50 ng/mL of RANKL), the RNA was extracted with High Pure RNA Isolation Kit (Roche, Basel, Switzerland). Briefly, 2 µg of RNA were used for retro-transcription using the High-Capacity cDNA Reverse Transcription Kit (Applied Biosystems, Foster City, CA, USA), followed by RT-qPCR with LightCycler^®^ 480 Probes Master (Roche) in a LightCycler480 following the commercial protocol. Primers for *Rank*,* Trap*,* Nfatc1*,* and Ctsk* were designed from available mouse sequences using the primer analysis software Primer3 (Table S1). The *Gapdh* gene was used as an internal control. The comparative threshold (Ct) method was used for data analysis expressed as 2-ΔCt. Three independent experiments were carried out for each biomarker.

### TRAP Colorimetric Assay

To evaluate osteoclast differentiation, cleaved tartrate-resistant acid phosphatase (TRAPcP) activity was examined. RAW wt and RAW-*Gba1*KO cells were seeded at a density of 25,000 cells/cm^2^ in 96-well plates with MEM-alpha medium without phenol red (Gibco) containing 1% FBS, 1% penicillin/streptomycin, and 50 ng/mL of recombinant murine RANKL (PeproTech). Conditioned media were collected at different times (0, 2, 4, 6, and 8 days post differentiation), and TRAPcP activity was colorimetrically measured by adding 18 mg/mL p-nitrophenyl phosphate (Sigma Aldrich) in the presence of 46 mg/mL sodium tartrate, ascorbic acid 8.8 mg/mL and a reaction buffer containing 1 M acetate, 0.5% triton X-100, 1 M NaCl and 10 mM EDTA (pH 5.5). After 15 min of incubation at 37°C, the reaction was stopped with 0.3 N NaOH buffer and immediately measured at 450 nm using a Glomax Multi+ luminometer (Promega, Madison, WI, USA). Three independent replicates were carried out.

### Bone Resorption Activity

Bone resorption activity was measured in undifferentiated RAW-WT and RAW-Gba1KO cells and after inducing differentiation with 50 ng/uL RANKL for 7 days. Cells were seeded at a density of 2500 cells/cm^2^ in a 96-well plate fluorometric bone resorption assay kit (KBRA-S96KIT, Cosmo Bio Co.) following manufacturer instructions. Data are expressed as mean ± SD of eight independent biological replicates.

### Western Blot

In order to evaluate expression levels of glucocerebrosidase (GCase) and cathepsin K, cells were washed once with PBS and lysed in RIPA buffer using cell scrapers. The protein extracts were quantified using the PierceTM BCA Protein Assay Kit (ThermoFisher Scientific, Waltham, MA, USA). Proteins (20 µg/lane, under denaturing conditions) were separated by electrophoresis in an SDS-polyacrylamide gel (SDS-PAGE, 12%) and transferred to PVDF membrane (Merck-Millipore, Burlington, MA, USA). We used the appropriate primary antibody against GCase/GBA 2 µg/mL (GBA 2E2 WH0002629M1, Sigma-Aldrich, St. Louis, MO, USA) and cathepsin K 1:1000 (Cell Signaling, Beverly, MA, USA), and antibody against actin 1:200 (A2066, Sigma-Aldrich) or α-tubulin T5168 1:4000 (Sigma-Aldrich) was used as loading controls. The membranes were developed using a peroxidase-conjugated secondary antibody anti-rabbit IgG (A0545) 1:10000 for GCase/GBA, cathepsin K and actin antibodies and anti-mouse IgG (A5906) 1:10000 for α-tubulin antibody (Sigma-Aldrich). A total of 3 experiments were performed for each biological replica.

### Statistical Analysis

Statistical analyses were performed using the GraphPad Prism v8 (GraphPad Software, San Diego, CA, USA), applying unpaired, paired t-tests, Mann–Whitney-U test or ANOVA as appropriate. Data are expressed as mean ± SD and are representative of three or more independent experiments.

## Results

### Generation of Gba1 Gene Knockout in an Osteoclast-Like Cell Line

**Fig. 1:**
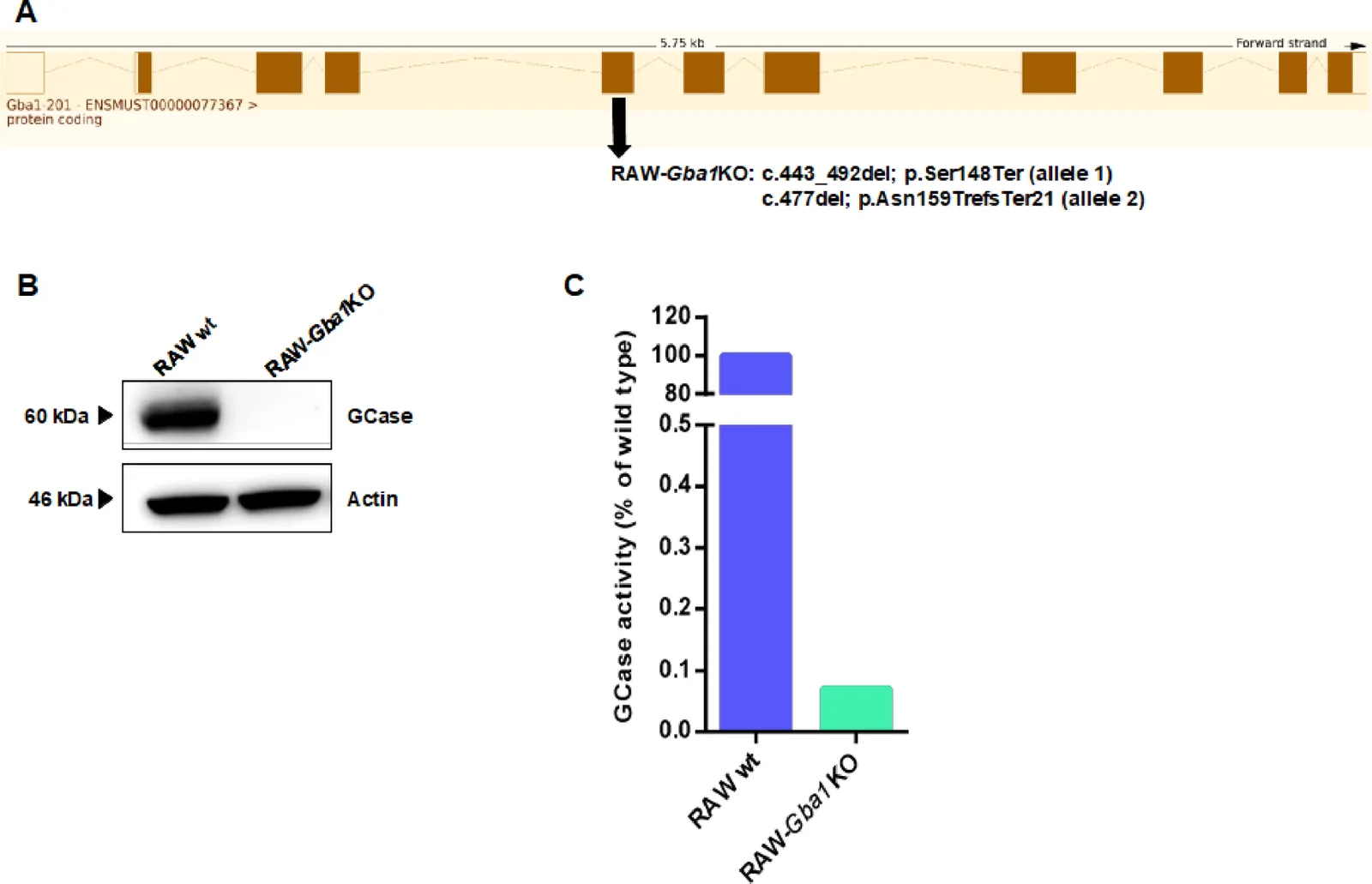
Generation and characterization of Gaucher cell models. (**A**) Schematic representation of *Gba1* gene from *Mus musculus* with the localised mutations. In this case we obtained compound heterozygous with 50 nucleotide deletions (443–492) on allele 1 and one nucleotide deletion (477) on allele 2. (**B**) WB analysis of glucocerebrosidase (GCase) expression in RAW cells (wt and KO). The KO clone did not showGCase expression. (**C**) GCase enzymatic activity of RAW cells (wt and KO) measured using the fluorogenic substrate 4-methylumbelliferyl–d-glucopyranoside. The fluorescent product was quantified in a fluorimeter at an excitation wavelength of 365 nm and emission of 495 nm. Quantification was performed in comparison to a reading without adding substrate to the cells (blank). Readings lower than the blank are shown as 0. The results correspond to one biological replicate for each cell type. A minimal signal was detected in the KO clone corresponding to the background derived from fluorescent reagent

We set out to generate and characterize *Gba1* knockout murine macrophage cell lines (RAW-*Gba1*KO) as putative osteoclastic cell models of GD. We obtained a single KO clone (RAW-*Gba1*KO), compound heterozygous for two frameshift deletions, one encompassing 50 nucleotides (NM_008094.7: c.443_492del; p.Ser148Ter) and another affecting 1 nucleotide (NM_008094.7: c.477del; p.Asn159TrefsTer21) (Fig. 1A). These deletions were validated by Sanger sequencing (Supplementary Figure S1).

The RAW-*Gba1*KO cells showed no GCase protein expression, as determined by western blot (Fig. 1B), and displayed less than 1% residual signal in the measurement of GCase activity (Fig. 1C).

### Functional Characterization of the Generated Osteoclastic Cells

**Fig. 2:**
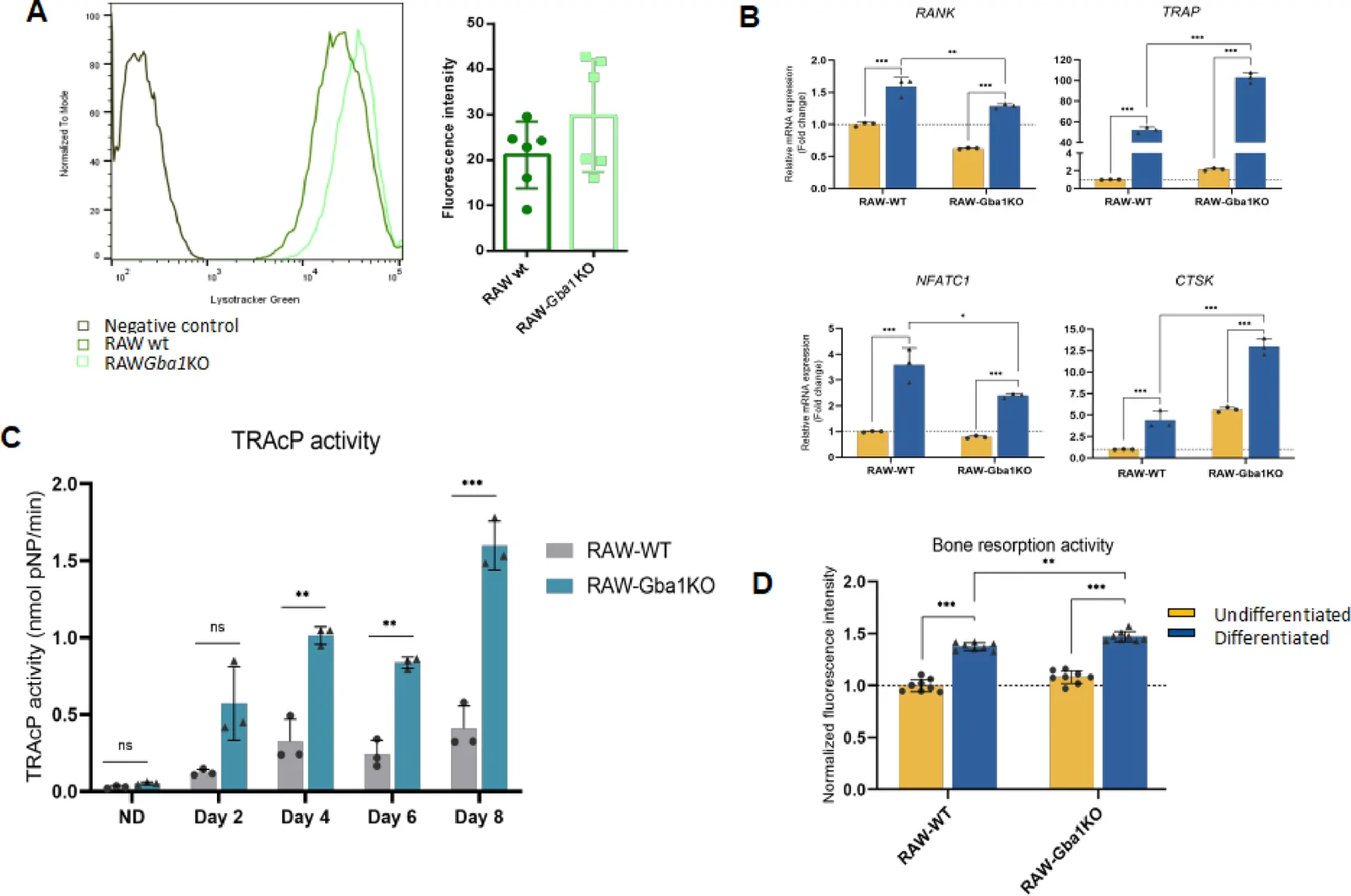
Characterization of the RAW-*Gba1*KO cell model. (**A**) Determination of lysosome status by flow cytometry using Lysotracker Green. The graph (right) corresponds to the mean fluorescence intensity values of 10 thousand events. Representative fluorescence intensity histograms for each cell type are shown inside the box (left panel). The results are expressed as mean ± SD of six independent experiments. (**B**) Gene expression of osteoclast differentiation markers in RAW-WT and RAW-Gba1KO cells. qPCR analysis of *Rank*,* Trap*,* Nfatc1*, and *Ctsk* mRNA in RAW-WT and RAW-Gba1KO cells, either undifferentiated or differentiated into osteoclast with RANKL for 8 days. Expression was normalized to *Gapdh* gene expression and is shown as fold change relative to the RAW-WT undifferentiated control. Data are expressed as mean ± SD of three independent biological replicates. Statistical significance was assessed by two-way ANOVA followed by Sidak’s multiple comparisons test, comparing undifferentiated vs. RANKL+ within each genotype and RAW-WT vs. RAW-Gba1KO within the RANKL+ condition (* *p* < 0.05; ** *p* < 0.01; *** *p* < 0.001). (**C**) TRAcP enzyme activity colorimetric assay in conditioned media from RAW264.7 WT and RAW264.7 Gba1KO cells following induction of osteoclast differentiation with 50ng/uL RANKL. Enzyme activity was detected in a non-differentiated control (ND), and at day 2, 4, 6, and 8 of differentiation. Results are expressed as mean ± SD of three independent biological replicates per condition. Statistical significance between RAW-WT and RAW-Gba1KO in the RANKL-treated group was assessed by Welch’s unpaired t-test at each timepoint (ns, *p* ≥ 0.05; * *p* < 0.05; ** *p* < 0.01; *** *p* < 0.001). (**D**) Bone resorption activity of RAW-WT and RAW-Gba1KO cells. Bone resorption was measured in RAW-WT and RAW-Gba1KO cells, either undifferentiated or induced toward osteoclast differentiation with 75 ng/uL RANKL for 7 days, using a fluorometric bone resorption assay kit (KBRA-S96KIT, Cosmo Bio Co.). Data was normalized against the undifferentiated control in each experiment and is expressed as the normalized mean ± SD of eight independent biological replicates. Statistical significance was assessed by two-way ANOVA followed by Sidak’s multiple comparisons test, comparing Undifferentiated vs. Differentiated within each clone and RAW-WT vs. RAW-Gba1KO (** *p* < 0.01; *** *p* < 0.001)

We first performed an analysis of the basal state of lysosomes in the generated osteoclast-like cells. An increased trend was observed in the fluorescence intensity of LysoTracker green of KO cells compared to wt cells (Fig. 2A).

Next, we determined the mRNA expression of four specific osteoclast biomarkers, *Rank*,* Trap*, *Nfatc1*, and *Ctsk* (cathepsin K) in the basal state and after inducing osteoclast differentiation for 8 days (Fig. 2B). As expected, we observed a statistically significant increase in the expression level of all markers after osteoclastic differentiation. *Rank* and *Nfatc1*, two master factors required for osteoclast differentiation, were significantly less expressed in differentiated RAW-*Gba1*KO cells compared to wt cells. Hence, osteoclastic differentiation seems reduced or delayed in KO cells. However, regarding *Trap* and *Ctsk*, RAW-*Gba1*KO cells showed higher expression levels compared to wt suggesting increased osteoclast activity. We performed a colorimetric assay to check the activity of TRAP protein (TRAPcP: active form of TRAP after proteolytic cleavage by cathepsin K) secreted to the culture media, using p-nitrophenyl phosphate as a substrate at different time points during osteoclast differentiation. We observe an increase of TRAPcP activity during the differentiation process in both cell types although this increase was significantly higher in the KO than in wt. Hence, from 4 days of osteoclastic differentiation, an excess of TRAPcP activity was detected in RAW-*Gba1*KO in comparison to wt cells (Fig. 2C).

Afterward, bone resorption was assessed by measuring fluorescence released to the medium in the action of osteoclast pit formation. Both lines increased bone resorption after 7 days of differentiation even though RAW-*Gba1*KO cells showed a significant increase in resorption activity (Fig. 2D).

**Fig. 3:**
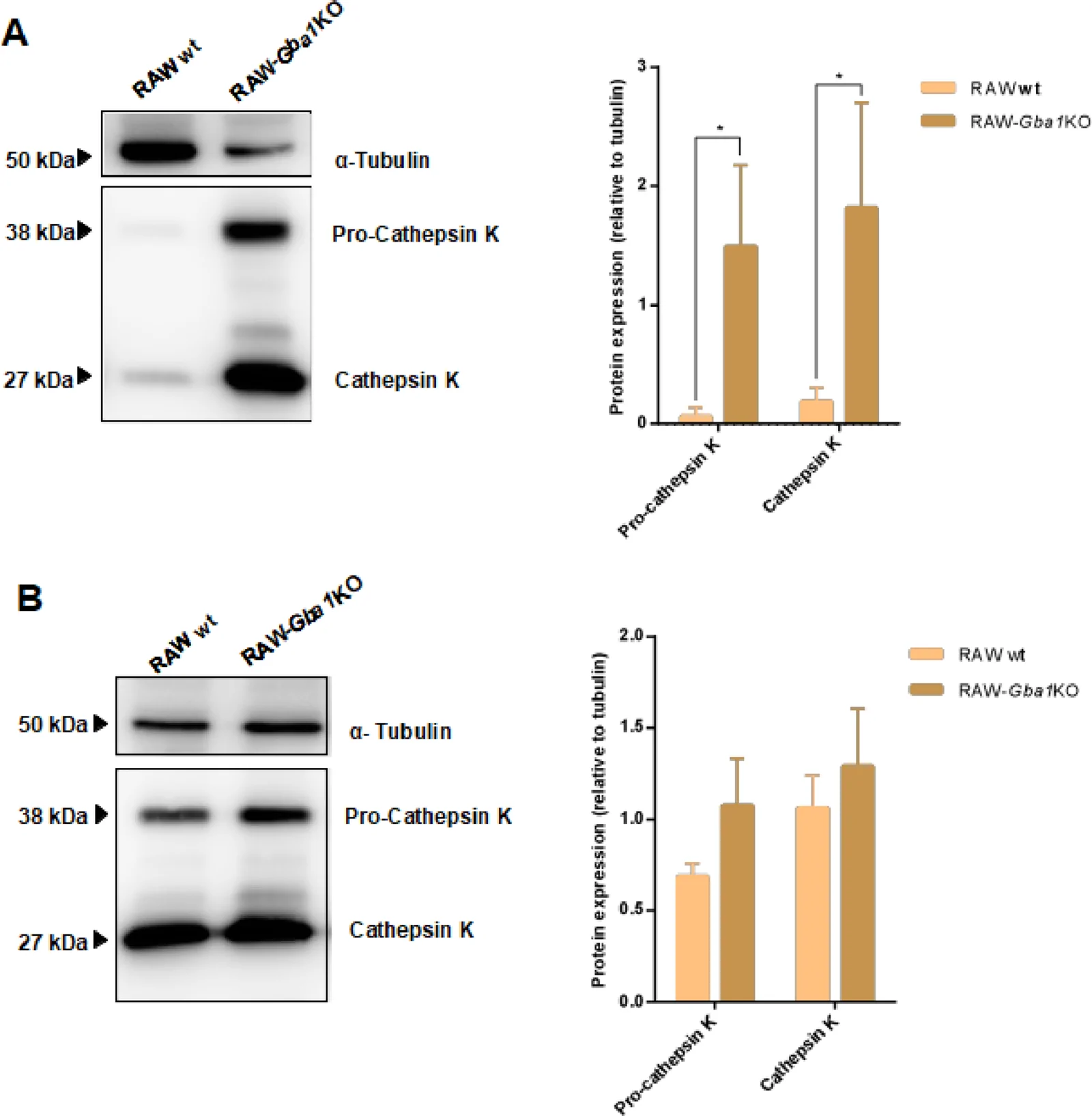
WB to analyze the expression level of pro-cathepsin K and cathepsin K before (**A**) and after (**B**) inducing differentiation to osteoclasts with 50ng/mL RANKL for 4 days. The intensity of the bands was quantified using the Fiji software and pro-cathepsin K and cathepsin K measurements were normalized with tubulin. Results are expressed as mean ± SD of three (**A**) or two (**B**) independent experiments. Statistical significance was assessed by unpaired t test, comparing RAW-WT vs. RAW-Gba1KO undifferentiated and differentiated. (* *p* < 0.05; ** *p* < 0.01)

Finally, we wanted to assess whether cathepsin K, the main activator of TRAP by proteolytic cleavage, was increased in KO cells compared to wt cells. Western blot analyses before inducing osteoclast differentiation showed strongly increased expression of both cathepsin K and pro-cathepsin K in the KO compared to wt cells (Fig. 3). After inducing osteoclast differentiation, we still observe increased protein levels in KO lines although differences were not statistically significant (Fig. 3).

## Discussion

Here we aimed to generate and characterise an osteoclast-like cellular model of GD to understand the mechanisms underlying the skeletal symptomatology associated with this pathology. Thus, we used CRISPR/Cas9 gene editing to generate *Gba1* knockouts in Raw cell lines. The use of rodent cell lines was preferred to human cells as they do not harbor the *GBA* pseudogene. After validating the Knockout model, we characterised it by analysing lysosomal state and determining the expression of osteoclastic biomarkers. Moreover, the osteoclast functionality was also tested analysing TRAP activity and bone resorption capacity.

Previous evidence supports a key role for osteoclasts in the bone pathology of Gaucher disease (GD). Early work by Moran et al. [13] reported elevated plasma cathepsin K activity in GD patients, while subsequent studies demonstrated that increased cathepsin K expression correlates with the severity of osteopenia [14]. Cathepsin K is a lysosomal cysteine protease predominantly expressed by osteoclasts and is essential for the degradation of bone matrix proteins during bone resorption. It is synthesized as an inactive proenzyme and undergoes intracellular processing within the lysosomal compartment before being released in its mature, active form [15, 16].

Consistent with these observations, our Gaucher osteoclast model (RAW-*Gba1* KO) exhibited marked overexpression of both pro-cathepsin K and mature cathepsin K. Notably, this increase was detected even prior to osteoclast differentiation and in the absence of any external induction. Furthermore, elevated expression persisted in differentiated osteoclasts, suggesting that *Gba1* deficiency intrinsically promotes cathepsin K upregulation. Interestingly, this occurred despite only a modest trend toward lysosomal accumulation, indicating that cathepsin K dysregulation may represent an early and robust feature of Gaucher osteoclast dysfunction.

Likewise, in our osteoclast-like *Gba1*KO cell model we observed enhanced tartrate-resistant acid phosphatase (TRAPcP) activity along differentiation when compared to wt cells, suggesting a potential role in Gaucher bone disease. TRAP is an enzyme that is highly expressed by osteoclasts and is involved in osteopontin/bone sialoprotein dephosphorylation. Its detection currently serves as a marker of osteoclast number and bone resorption and indeed, cytochemically detected TRAP expression has been used in Gaucher’s disease as a disease-associated marker since the early days [17]. We observed a significantly higher *Trap* mRNA expression in RAW-*Gba1*KO cells during osteoclast differentiation consistent with the enhanced TRAP enzyme activity detected in RAW-*Gba1*KO cells. Reithmeier et al. [18] have recently demonstrated the direct action of cathepsin K in the cleavage of the low activity TRAP5a to produce the TRAP5b isoform, with high phosphatase activity. This isoform has been proposed as a potential bone biomarker for GD with the capacity to predict the progression of bone density abnormalities [19]. In consequence, high levels of active cathepsin K in RAW-*Gba1*KO cells could be also involved in the enhanced TRAP activity. All of this is an intrinsic characteristic of the Gaucher osteoclast that may play a direct role in bone disease.

Otherwise, when osteoclastic differentiation markers (*Rank* and *Nfatc1*) were analyzed, RAW-*Gba1*KO cells showed decreased expression levels compared to wt cells, suggesting a lesser or delayed differentiation. However, these “less-differentiated” osteoclasts are more active than differentiated RAW wt cells when analyzing *Trap* and Cathepsin K both at gene expression and protein level. This is corroborated by the increased bone resorption capacity detected in the KO cultures.

Our Gaucher cell model has several limitations, primarily related to its origin as a transformed cell line and its maintenance as an isolated monoculture. These characteristics preclude the study of cell-cell interactions and the complex physiological processes that occur in vivo, potentially limiting its ability to fully recapitulate Gaucher disease pathology. Nevertheless, the model is easy to maintain and provides a valuable tool for investigating cell-autonomous mechanisms that may contribute to the skeletal phenotype of the disease.

Skeletal involvement represents a major source of morbidity in GD, substantially impairing quality of life through pain, disability, and functional limitations. Importantly, bone complications can develop regardless of disease onset, genotype, or the severity of visceral and hematological manifestations. Despite the clinical relevance of skeletal disease, only 14% of studies on bone manifestations in GD are experimental, highlighting a significant gap in the field [20]. This underscores the need for further mechanistic research, continuous evidence updates, and broader dissemination of findings to improve patient management and facilitate the development of novel therapeutic strategies.

## Conclusion

**Fig. 4:**
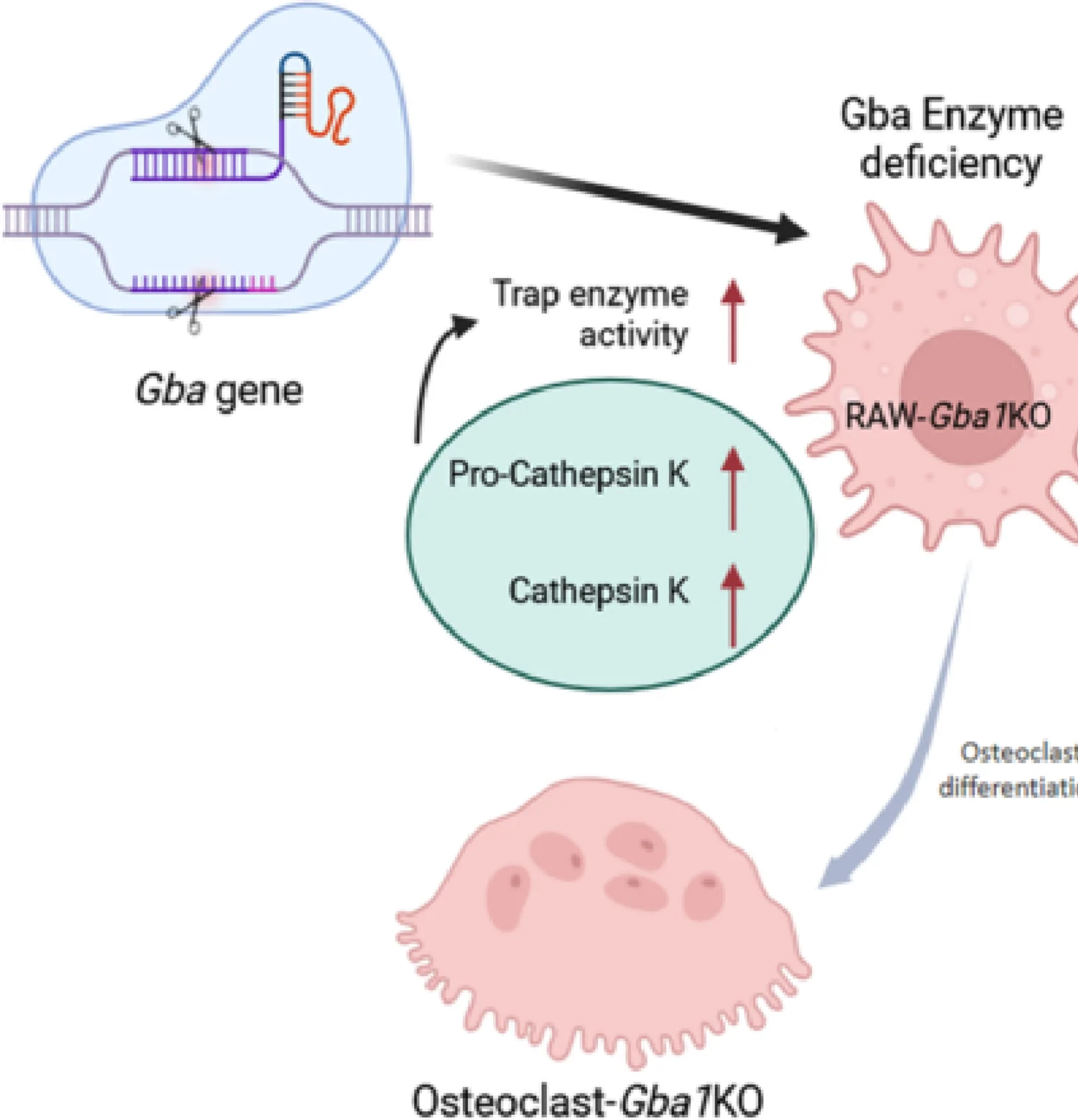
Schematic representation of the generated and characterized cell model. Our results suggest an intrinsic osteoclastic defect. The red arrows indicate the biomarkers that favors bone resorption

In conclusion, in the context of our cell model, our findings point out an intrinsic osteoclastic defect in Gaucher disease (Fig. 4). This could suggest that osteoclasts would be the main drivers of bone pathology, reinforcing the evidence obtained from various in vitro and in vivo models by other authors.

## Supplementary Information

Below is the link to the electronic supplementary material.

Supplementary Material 1

Supplementary Material 2

## Data Availability

Data is contained within the article or supplementary material. Other raw data are available upon request to the corresponding author.
