# Supplementary material for: Generation and Characterization of an Osteoclastic Cell Model of Gaucher’s Bone Disease
Source: Calcif Tissue Int. 2026 Oct 1;117(1):154. doi: 10.1007/s00223-026-01616-5 (PMC13627245; doi:10.1007/s00223-026-01616-5)
Supplement: Supplementary file 2 [file 223_2026_1616_MOESM2_ESM.docx]

**Supplementary Material**

**Supplementary Figure S1:** RAW-*Gba1*KO cells. Deletion of 50 nucleotides on allele 1 (upper panel) and 1 nucleotide on allele 2 (lower panel).

**Supplementary Figure S2:** Giemsa staining in RAW wt (A) and RAW-Gba1KO (B) cells undifferentiated, and differentiated cells with Rankl 50ng/mL for four days (C) and (D) respectively. White arrows indicate osteoclasts (cells with 3 or more nuclei). The right panels show in detail an osteoclast for each cell type indicated by the red arrow. Scale bar: 100μm

**Supplementary Figure S3:** RAW wt and RAW-Gba1KO cells differentiated with Rankl 50ng/mL for eight days. Scale bar: 500μm

**Supplementary Table S1:** Primers used for RT-PCR analyses. Primers were designed from available mouse and rat sequences using the primer analysis software Primer3. Sigma-Aldrich Synthetic guide RNA (RN00064-0684536) is shown in the last file.

| **NAME** | **ORGANISM** | **SEQUENCE** |
| --- | --- | --- |
| *Gapdh* (Fw) | Mouse | 5'-ACCATCATCCCTGCCTCTAC-3' |
| *Gapdh* (Rv) | Mouse | 5'-CCTGTTGCTGTAGCCAAAT-3' |
| *Rank* (Fw) | Mouse | 5'-TGGGTCAATGATGCTTGCAG-3' |
| *Rank* (Rv) | Mouse | 5'-ATCTCTGACTTCTGCCCAGG-3' |
| *Trap* (Fw) | Mouse | 5'-CACGAGAGTCCTGCTTGTC-3' |
| *Trap* (Rv) | Mouse | 5'-AGTTGGTGTGGGCATACTTC-3' |
| Nfatc1 *(Fw)* | Mouse | 5'-CTCAGGCCCGTATGAGCTTC-3' |
| Nfatc1 *(Rv)* | Mouse | 5'-GGAGTGGGATCTCCAGGACT-3' |
| Ctsk *(Fw)* | Mouse | 5'-GCAGAACGGAGGCATTGACT-3' |
| Ctsk *(Rv)* | Mouse | 5'- CCTCTGCATTTAGCTGCCTTTG-3' |
| sgRNA (RN00064-0684536) | Mouse | 5'-CTTCTGGCTGCAAGGTTAGT-3' |

**Supplementary Table S2:** Number of off-targets according to results from Benchling

| ***In silico* Off-target Prediction** | | | | |
| --- | --- | --- | --- | --- |
| **Sequence** | **PAM** | **Score** | **Gene** | **Cut locus** |
| CTTCTGGCTGCAAGGTTAGT | AGG | 100 | *Gba1 (ENSRNOT00065043154.1)* | chr2:-188517808 |
| **ATGCTGGCAGCAAGGTTAGT** | AAG | 1,7 |  | chr13:-28677348 |
| CT**A**C**A**G**A**CTGCAAGGTTAGT | AAG | 1,7 |  | chr4:-97570655 |
| **GTGCTGGCTGCAAGGTGAGT** | CAG | 1,5 |  | chr1:+210265431 |
| **GTACAGGCTTCAAGGTTAGT** | GAG | 1,4 |  | chr18:-24099113 |
| CTTCTG**TT**TG**G**AAGGTTAGT | GAG | 0,9 |  | chr17:+26056386 |
| C**AA**CT**A**G**A**TGCAAGGTTAGT | CAG | 0,9 |  | chr2:+116093750 |
| **GTTCAGGCTCCAAGGTTAGG** | AAG | 0,8 |  | chr2:-140859102 |
| C**G**TC**CT**GCT**C**CAAGGTTAGT | GGG | 0,8 |  | chr3:-165367006 |
| **ATTAAGGCTGCAAGGTCAGT** | TGG | 0,7 |  | chr12:-9999542 |
| C**C**TCTGG**T**T**C**C**T**AGGTTAGT | TGG | 0,7 |  | chr14:+87717959 |
| C**C**TCTGG**T**T**C**C**T**AGGTTAGT | TGG | 0,7 |  | chr14:+87705942 |
| C**CC**CTGG**G**TGCA**G**GGTTAGT | AAG | 0,7 |  | chr13:+35309737 |
| CT**G**CTGG**T**T**T**CAAGGT**C**AGT | CAG | 0,6 |  | chr14:-45416799 |
| C**AG**CT**TC**CTGCAAGGTTAGT | CAG | 0,6 |  | chr8:-110550471 |
| **AATCTGCCTGCCAGGTTAGT** | AGG | 0,6 |  | chr5:+127430438 |
| **TTTCTGGATGCAAAGTTAGT** | CAG | 0,5 |  | chr9:+2028467 |
| **TTTCTAGCTACAAGGTTAGG** | CAG | 0,5 |  | chr19:-14105711 |
| CTTCTG**C**CTG**GG**AGGTTAGT | GAG | 0,5 |  | chr10:+108991200 |
| C**AG**CT**A**GCTGC**T**AGGTTAGT | TGG | 0,5 |  | chr4:-67188444 |
| **GTTGTGGCTGATAGGTTAGT** | CGG | 0,5 |  | chr3:+38534096 |
| **TATCTGCCTGCATGGTTAGT** | GAG | 0,5 |  | chr2:-158151678 |
| **ATTATGGCTGGAAGGTGAGT** | TAG | 0,4 |  | chr1:+209272758 |
| **TTTCTGGGTGAAAGGTCAGT** | TGG | 0,4 |  | chr2:+234433883 |
| **TTTCTGGGTGAAAGGTCAGT** | TGG | 0,4 |  | chr2:-234237600 |
| CT**G**C**CT**GCTGCAAGGT**C**AGT | AGG | 0,4 |  | chr3:-176480538 |
| CT**C**CT**T**GCT**T**CAAGGT**C**AGT | GGG | 0,4 |  | chr3:+163624146 |
| CTTCTG**CT**T**C**CAAGGT**G**AGT | GGG | 0,4 |  | chr7:-119346141 |
| **GATCTGGCTGCATGGTTAGA** | TGG | 0,4 |  | chr1:-17564832 |
| **ATTATGGCTGTAAGGTTAAT** | AAG | 0,4 |  | chrX:+129546514 |
| C**A**T**T**TGGCTGCAAGGT**A**AG**A** | GAG | 0,4 |  | chr10:+82972034 |
| **TTTCTAGCTCCATGGTTAGT** | CAG | 0,4 |  | chr1:-159528888 |
| CTTC**CC**GCT**T**CAAGGT**G**AGT | GGG | 0,4 |  | chr3:+61666190 |
| CTT**G**T**A**G**G**TGCA**G**GGTTAGT | GGG | 0,4 |  | chr20:-4273214 |
| CTT**G**T**A**G**G**TGCA**G**GGTTAGT | GGG | 0,4 |  | chr20:-2934936 |
| CTT**G**T**A**G**G**TGCA**G**GGTTAGT | GGG | 0,4 |  | chr20:-2912491 |
| CTT**G**T**A**G**G**TGCA**G**GGTTAGT | GGG | 0,4 |  | chr20:-2880845 |
| CTT**G**T**A**G**G**TGCA**G**GGTTAGT | GGG | 0,4 |  | chr20:-2850035 |
| CTT**G**T**A**G**G**TGCA**G**GGTTAGT | GGG | 0,4 |  | chr20:-2816344 |
| CTT**G**T**A**G**G**TGCA**G**GGTTAGT | GGG | 0,4 |  | chr20:-2793993 |
| CTT**G**T**A**G**G**TGCA**G**GGTTAGT | GGG | 0,4 |  | chr20:-2760022 |
| CTT**G**T**A**G**G**TGCA**G**GGTTAGT | GGG | 0,4 |  | chr20:-2726244 |
| C**A**TCTGGCTGCAAG**T**TTA**C**T | GAG | 0,4 |  | chr8:+52224385 |
| CT**GA**TGGCTG**A**A**G**GGTTAGT | CAG | 0,4 |  | chr8:-47194267 |
| CTT**AA**GGCTGCAAGGT**A**AG**A** | TGG | 0,3 |  | chr6:-130881941 |
| **ATTATGGCTGCCTGGTTAGT** | CGG | 0,3 |  | chr20:+12275063 |
| C**A**TCTG**T**CTGCAA**A**GTTAGT | GAG | 0,3 | Nhlh1 (ENSRNOG00000005166) | chr13:+90442953 |
| CTT**TG**GGCTG**G**AAGGTTA**C**T | TGG | 0,3 |  | chr14:-13063002 |
| CTTC**A**G**A**C**C**G**A**AAGGTTAGT | AGG | 0,3 |  | chr18:-2045928 |
| C**G**TCTGGCT**C**C**C**AGGT**G**AGT | CAG | 0,3 |  | chr17:+54583323 |
